# Using the diffusion of innovations theory to assess socio-technical factors in planning the implementation of an electronic health record alert across multiple primary care clinics

**DOI:** 10.14236/jhi.v23i1.157

**Published:** 2016-04-15

**Authors:** Ching-Ping Lin, Janelle Guirguis-Blake, Gina A. Keppel, Sharon Dobie, Justin Osborn, Allison M. Cole, Laura-Mae Baldwin

**Affiliations:** Institute of Translational Health Sciences, University of Washington, Seattle, USA; Tacoma Family Medicine Residency Program, Tacoma, USA; Institute of Translational Health Sciences, University of Washington, Seattle, USA, and Department of Family Medicine, University of Washington, Seattle, USA; Department of Family Medicine, University of Washington, Seattle, USA; Department of Family Medicine, University of Washington, Seattle, USA; Institute of Translational Health Sciences, University of Washington, Seattle, USA, and Department of Family Medicine, University of Washington, Seattle, USA; Institute of Translational Health Sciences, University of Washington, Seattle, USA, and Department of Family Medicine, University of Washington, Seattle, USA

**Keywords:** adverse drug event (ADE), chronic kidney diseases (CKDs), electronic health records (EHRs), medication alert systems, outpatient clinic, primary care

## Abstract

**Background:**

Adverse drug events (ADEs) are a leading cause of death in the United States. Patients with stage 3 and 4 chronic kidney disease (CKD) are at particular risk because many medications are cleared by the kidneys. Alerts in the electronic health record (EHR) about drug appropriateness and dosing at the time of prescription have been shown to reduce ADEs for patients with stage 3 and 4 CKD in inpatient settings, but more research is needed about the implementation and effectiveness of such alerts in outpatient settings.

**Objective:**

To explore factors that might inform the implementation of an electronic drug–disease alert for patients with CKD in primary care clinics, using Rogers’ diffusion of innovations theory as an analytic framework.

**Methods:**

Interviews were conducted with key informants in four diverse clinics using various EHR systems. Interviews were audio recorded and transcribed.

**Results:**

Although all clinics had a current method for calculating glomerular filtration rate (GFR), clinics were heterogeneous with regard to current electronic decision support practices, quality improvement resources, and organizational culture and structure.

**Conclusion:**

Understanding variation in organizational culture and infrastructure across primary care clinics is important in planning implementation of an intervention to reduce ADEs among patients with CKD.

## INTRODUCTION

Medical errors are the eighth leading cause of death in the United States**^1^** and the most common form of medical error in inpatient settings, estimated at one medication error per patient per day.**^2^** Patients in ambulatory care settings are also subject to medication errors, with one study documenting an overall medication error rate of 7.6%. Adverse drug events (ADEs) result in increased morbidity and mortality, increased cost, and excessive healthcare utilization.**^2–4^**

Medication dosing errors and toxicity are especially important and common problems in patients with chronic kidney disease (CKD).**^5,6^** The incidence of ADEs is much higher in patients with CKD than those without CKD.**^7^** Although standard dosing guidelines and methods of dose adjustment are available for patients with CKD,**^8^** between 5.3% and 69.6% of medications that require dose adjustment are dosed inappropriately for patients with CKD.**^9–12^**

Two main strategies have been tested, almost exclusively in the inpatient setting, to assist practitioners in monitoring and adjusting drug therapy among CKD patients: computerised dosing programs and clinical pharmacist dosing services.**^13,14^** Use of these strategies can decrease dosing errors and the prescription of high-risk medications.**^15–18^** We found only one study evaluating a computerised tool in the outpatient setting. Its results suggest that alerting outpatient pharmacists to possible errors in drug selection and dosing at the time of dispensing can decrease medication errors.**^19^**

Computerised physician order entry (CPOE) is increasingly used in ambulatory clinics to improve medication safety and quality of care.**^20^** With the advent of incentive programs tied to meaningful use of an organization’s electronic health record (EHR), including effective decision support, a realistic strategy to improve medication management is to develop evidence-based alerts in ambulatory care that build on CPOE systems. An important next step is to evaluate whether this strategy can decrease dosing errors and other medication errors in the ambulatory care setting.

This study explored how best to implement electronic drug–disease alerts for patients with CKD in four primary care clinics in the Northwest United States. Electronic drug–disease alerts are computerised warnings at the time of prescription that an EHR produces to alert a provider about the use of certain drugs in patients with specific conditions and diseases (Table 1). We recognized that working in a diverse set of community practices created heterogeneity of EHRs and numerous socio-technical factors. Research has documented that the process for planning implementation in a particular setting can be as important as the intervention itself.**^21^** We wanted to explore these socio-technical factors because we knew a single implementation strategy might not succeed equally across clinics. This project asked what key elements might increase the likelihood of success in implementing electronic drug–disease alerts for patients with CKD across diverse clinics.

## METHODS

### Practice sites and selection

This study was conducted in the Washington, Wyoming, Alaska, Montana, and Idaho (WWAMI) region Practice and Research Network (WPRN), a practice-based research network in the five-state WWAMI region. We selected four WPRN clinics for this study based on the maturity of their EHR implementations and the ability to query the EHR. Two clinics are located in large cities (population >100,000) and two in smaller rural-serving cities. One clinic is hospital-affiliated, one university-affiliated, and the other two are community health centers. The number of annual patient visits in the clinics ranges from roughly 17,000–50,000.

### Framework application

We used Rogers’ diffusion of innovations (DOI) theory framework for how new innovations are adopted by organizations and Greenhalgh’s subsequent work adapting the framework for healthcare settings.**^22,23^** We used these frameworks to deductively explore factors that might help the intervention better diffuse in each clinic setting. The DOI framework, developed originally from studies in rural sociology, has been used frequently in healthcare settings, such as to implement metabolic syndrome screening guidelines**^24^** and screening of mechanically ventilated patients for delirium.**^25^**

We focused on three of the four elements of the DOI framework most relevant to this study’s purpose: innovation, communication channels and social system (Table 2). Within innovation, we focused on three of the five characteristics of innovations that influence their adoption: compatibility, complexity and relative advantage.

### Interview guide and interview procedures

We developed an interview guide (Appendix 1) that focused on understanding each clinic’s EHR capabilities, current availability of CKD-related decision support, organizational culture, organizational structure, and research and quality improvement (QI) infrastructure. Two study team members (JGB and CPL) conducted seven interviews with key informants at the four clinics, one team member leading while the other took notes. At each clinic, we interviewed a care provider able to describe the current state of CKD decision support and the clinic’s culture. At three sites, we also interviewed an individual with specific knowledge of the clinic’s EHR system, as the care providers did not have the technological expertise to answer all interview questions. Four key informants were physicians, one a clinical pharmacist, one a director of quality, and one both a physician and director of clinical quality and population management.

Six interviews were conducted in person, one by phone. All were recorded and transcribed. We gathered over 225 min of interviews resulting in 92 transcribed pages. Participants reviewed summaries of the interviews and corrected, clarified and/or supplemented the data as appropriate.

### Analysis

The analysis mapped themes identified in the qualitative data to the DOI framework described above. Two coders (CPL and GK) developed a codebook based on the modified DOI framework. They used a deductive coding method starting with the DOI framework, then added open codes and reconciled them with the framework. Each coder separately coded interviews using ATLAS.ti.**^26^** The coders used an iterative process of reviewing and reconciling codes until agreement was reached. Other authors reviewed the primary coders’ themes and codes, verifying and augmenting them per their interpretation of the data. We received approval for this study from the University of Washington Human Subjects Division.

## RESULTS

### Innovation

#### Compatibility

We assessed compatibility (how well the innovation fits with the intended adopters’ values, norms and perceived needs) in three ways:

1. We compared the compatibility of the proposed intervention with the electronic alert capabilities at the clinics (Table 3). Of the four participating clinics, all had existing EHRs with the capability for drug–disease alerts, but none had implemented these alerts. Sites 1, 3 and 4 had electronic alerts active in their EHR systems. Site 3 had limited its alerts to high-priority drug–drug and drug allergy alerts.
2. We examined the clinics’ experience with and infrastructure for supporting research or QI projects. All four clinics had QI boards or processes. Sites 2 and 3 had dedicated QI staff and commonly conducted QI projects using EHR data.
3. We investigated whether the clinics intended to meet one of the stage 1 meaningful use core objectives by implementing drug–drug and drug allergy alerts. Meaningful use is a program that sets specific objectives that eligible professionals and hospitals must achieve to qualify for Centers for Medicare and Medicaid Services Incentive Programs.**^27^** Implementing drug–drug and drug allergy ‘interaction checks’ is a core objective for stage 1 meaningful use. Sites 2 and 3 had already met meaningful use criteria using methods that did not involve electronic alerts. Site 4 reported that alerts might be part of their meeting meaningful use criteria, but that their alerts would need to be reviewed in order to do so, saying: ‘we will probably reevaluate our alerts here relatively soon because we’re moving into meaningful use attestation… And my suspicion is that we will want to turn on a set of alerts for meaningful use. And it may be that in the process of doing that, we decide…we’d rather bother [providers] for pneumovax which is one of our requirements instead of this one’.

#### Complexity

Complexity is the degree to which the innovation is perceived as difficult to use. Alert fatigue, the process of receiving too many alerts, causing the receiver to ignore alerts,**^28^** emerged as a common concern among all the key informants interviewed: ‘So the biggest barrier that I can think of is…alert fatigue… we all see an alert virtually every single time we enter the EMR, of one kind or another and you know, this would be just one more alert. But I do think that at least at this point in time, there are not many of our alerts that are really patient oriented…*’*

Key informants also discussed the importance of the alert fitting into their workflows, or being ‘useful’, ‘relevant’ or ‘time saving’. For example, one participant shared, ‘I think the question is when it flags you. I know early on they turned on some things that popped up as soon as you logged in and those really weren’t that helpful. It needs to happen around the time you’re ordering’. Another noted, ‘I think it’s going to be a welcome function as long as it doesn’t complicate current prescribing practices. If they can be seamlessly integrated into [the EHR system] without multiple additional clicks, I think it would be a very welcome piece’. One participant gave an example of a new EHR feature that did not have these traits, ‘[there were] tons more buttons and they were in different locations… And while it gives more usability, it’s like shopping at Costco when you just want a small spice or something’.

#### Relative advantage

Relative Advantage is how well the innovation has a clear unambiguous advantage compared with existing strategies. Without electronic drug–disease alerts, providers are required either to know or to look up each medication at the time of prescribing to determine whether it requires dose adjustment or is contraindicated. This process generally involves several steps to access both the calculated glomerular filtration rate (GFR) from the chart and a database with information on medication renal dosing.

None of the clinics had CKD-related drug–disease alerts (Table 3). All of the clinics reported that they had a method to calculate GFR rates in the EHR or that laboratory reports return GFR calculations. Providers use this information at their discretion.

### Communication

We looked at communication channels to understand how change was effected in each organization. Each clinic had a different culture of communication (Table 3). Site 2 regularly used team meetings with providers and nursing staff to communicate new information or initiatives. However, this method did not fully reach all of their staff: ‘One of the biggest problems that we have…is it’s just really hard to get everybody in the room and let them know what’s happening, not to mention your nurses. I mean even today, we’ve got one nurse sick and one nurse who’s off and we’re having our discussion about using [a new practice management tool]. So you can never capture everyone*’*.

For Site 1, clear communication was critical to its culture, with an emphasis on training for new initiatives: ‘the local hospital…recently implemented the CPOE process there without any training at all, and the providers were kind of up in arms over that, you know, they were very clear about-do not do that here’.

Sites 3 and 4 had a more ‘hands off’ approach to communication. Site 3 primarily used email to communicate and did not require approval from providers prior to implementing EHR changes. At this clinic, providers were accustomed to learning about new functions through their everyday use of the EHR. Key informants from site 4 also reported that their clinic has no standard method for communicating change.

### Social system

We examined the social system at each clinic, focusing on how decisions were made, which is integral to how quickly an innovation will be adapted. The clinics that are members of larger health systems had top-down management and decision making, whereas the other clinics had much more local decision-making authority (Table 3). For instance, site 2 is an independent clinic whose information technology was managed on-site by clinic-employed personnel. Therefore, all of its EHR customizations and implementations could be conducted locally, including provider-level customizations. Site 2’s key informant shared an example of provider-level customization, ‘you open up one of Dr … ’s patients that he is designated as the PCP (primary care provider) on? It will say basically, …call me. Don’t monkey with my patients too much’*.*

In contrast, sites 3 and 4 are members of large health systems with centralized information technology systems, staff and committees. EHR-related decisions were made for the entire health care system rather than for individual clinics. Physicians from site 3 reported that they were accustomed to new features appearing in the EHR without their direct involvement in the decision-making process. Site 4’s organization had recently implemented a new policy that any changes to the EHR must be system wide, rather than on a clinic-by-clinic basis. ‘So in the early days we could build all our own templates, we could do whatever, and now there’s… We’re becoming the battleship that’s slowly turning and no longer little speedboats’.

Site 1 is the largest clinic in a small network of clinics. This clinic hosted administrative committees and thus had a lead role in making decisions about EHR changes. This clinic was both similar to site 2 in its ability to drive change at a local level and subject to some of the same collective decision-making policies of the larger health systems.

Clinical pharmacists are one resource to promote and support an alert-based intervention. All but one clinic employed a clinical pharmacist as an integral member of the clinical care team.

## DISCUSSION

### Principal findings

This study discovered that the proposed CKD drug–disease alert was variably compatible with resources and prescribing processes at four primary care clinics, which had a wide variety of organizational structures and communication cultures. It is critical that we tailor an implementation plan for each organization to these factors to maximize acceptance. The DOI framework can be used to guide the design of an implementation plan for a CKD electronic drug–disease alert.

### Implications of the findings

#### Compatibility with Clinic Values, Norms and Needs

There was substantial variability in the availability of electronic alerts in the four participating clinics. In a clinic without alerts, providers would not be accustomed to interruptions or stops to their prescribing workflow and the proposed intervention might represent a fundamental change, leading to potential disruption and rejection.**^29^** For these clinics, the implementation plan would need to be designed in a way that specifically addressed the introduction of electronic drug–disease alerts and any resulting shift in provider workflow.

One clinic had a robust QI infrastructure that could support long-term evaluation and follow-up. At the clinics that had limited QI resources, it would be critical to ensure that the necessary resources would be available for follow-up and monitoring CKD drug–disease alerts.

We thought that making the proposed intervention part of a larger QI effort such as meaningful use might be an incentive to potential partner clinics. However, we discovered that several had already met meaningful use criteria using methods that did not involve electronic alerts, and a third had other higher priority alerts to implement. Meaningful use standards may still serve as a possible incentive towards implementing an alert-based intervention, but clearly our approach must be tailored to each clinic.

#### Complexity of the intervention

This study found that the intervention must fit into a clinician’s existing workflow, saving time and being efficient. This reinforces the importance of making sure that an intervention can meet these requirements before engaging with the clinics. Additionally, when working with the clinics, we must evaluate the intervention using these criteria to show the clinics that we understand their priorities and to ensure better long-term acceptance. Our key informants made clear that an alert that would require significant workflow redesign at the level of the provider alert interface would serve as a barrier to its use.

#### Relative advantage compared with existing strategies

The proposed intervention would use the available GFR data in the EHR to identify patients with CKD and, for these patients, alert the provider with a pop up window at the time that a contraindicated drug or inappropriate drug dose was prescribed. The pop up window would support the provider by warning about drug contraindications for patients with CKD and by recommending drug doses or frequencies that fit the patient’s level of kidney function. This introduces new clinical functions (e.g. identification of patients previously not identified with CKD and recommended changes in drug dosing based on GFR) and a new delivery method for this information (pop up window). Thus, the proposed intervention would add new capabilities and necessitate culture change at each of the clinics, since there were no existing drug–disease alerts. It would be critical to emphasize the benefit of this new type of alert and the advantage over current methods to the clinics and their providers.

#### Communication channels and social system

Understanding clinics’ existing communication and social systems allows development of individualized communication strategies for each setting. We discovered two general types of staff communication channels: face-to-face all-staff meetings and communication to staff via electronic means such as emails. Thus, an implementation plan for an alert-based intervention would best include support for multiple communication and information dissemination methods. This study suggests that different sites may be more accustomed to or expect use of certain communication channels. Supporting multiple approaches should provide the needed flexibility to respect clinic culture when choosing information dissemination methods while being sensitive to the information overload already facing clinicians.

### Comparison with Previous Research

Like this study, Bates et al.**^30^** found that an intervention must fit into the existing workflow, saving time, being efficient and changing direction rather than forcing a hard stop in work. They also recommend continuous maintenance of alerts to reflect current evidence and to monitor the usage pattern of alerts. Though alerts can be an important tool for improving drug safety,**^31^** Cho et al.**^32^** have demonstrated that providers do not heed (‘override’) the recommendations in alerts for renal dosing of medications most of the time, and only about 30% of these overrides are appropriate. A small number of providers were responsible for a large proportion of the overrides. This suggests that alerts must be accompanied by other interventions to ensure success. Kawamoto et al.’s**^33^** systematic review of trials using clinical decision support systems to improve clinical practice identified features that predicted the success of these systems, including integration into clinician workflow at the time and location of decision making and provision of recommendations rather than assessments only.

### Call for Further Research

Electronic drug–disease alerts have the potential to include the features that Kawamoto et al.**^33^** identified as predictive of success, yet additional research is needed to directly test the effectiveness of individual alert features. Practice-based research networks that include multiple clinics with EHR capabilities, sometimes within larger health systems, provide a robust environment in which to implement experiments of these features.**^34,35^**

### LIMITATIONS OF THE STUDY

We interviewed at most two key informants at each clinic and thus did not receive multiple perspectives from the same organization. Because this preliminary research did not focus on the willingness of the clinics to engage in implementing this intervention, but rather on the socio-technical characteristics of each setting, we felt that these informants could accurately represent their clinics.

## CONCLUSION

Rogers’ DOI theory provided a useful framework for exploring factors that might influence the implementation of a CKD drug–disease alert in four community-based primary care clinics. Rogers describes organizational culture as ‘how things are done’. This work shows that how things are done in community-based primary care settings can vary greatly, especially in terms of decision making and communication to providers. Given these findings, it is not surprising that clinics that are provided practice facilitators to assist with tailored clinical redesign and QI are significantly more likely to change their clinical practices than clinics without facilitators.**^36,37^** Those who implement new interventions in these settings, whether informatics based or not, must be conscious of clinic differences, understand how they might impact their implementation planning and tailor the interventions to different clinical settings in order to maximize the chances of acceptance.

## Figures and Tables

**Table 1:** Glossary of acronyms and terms

Acronyms/Terms	Definition
ADE	Adverse drug event
CKD	Chronic kidney disease
CPOE	Computerised physician order entry
Drug-allergy alert	A computerised warning at the time of prescription that an EHR produces to alert a provider about the potential for an adverse event related to the drug being prescribed and any allergies or adverse drug reactions recorded in the EHR.
Drug-disease alert	A computerised warning at the time of prescription that an EHR produces to alert a provider about the use of certain drugs in patients with specific conditions and diseases.
Drug-drug alert	A computerised warning at the time of prescription that an EHR produces to alert a provider about the interactions between the drug being prescribed and other drugs on the patient’s active medication list.
Electronic alert	Any computerised warning at the time of prescription, including drug-disease alerts, drug-drug alerts and drug allergy alerts.
QI	Quality improvement

**Table 2:** Conceptual framework based on Rogers’ DOI theory

Elements of DOI Framework	Definition
Innovation	A novel set of behaviors, routines and ways of working that are directed at improving health outcomes.
	*Compatibility*
	The degree to which the innovation is compatible with the intended adopters’ values, norms and perceived needs.
	*Complexity*
	The degree to which key players perceive the innovation as simple to use.
	*Relative advantage*
	The degree to which the innovation has a clear, unambiguous advantage to existing strategies
Communication channels	The channels by which an innovation is spread.
Social system	The system in which the innovation is spread.

**Table 3:** Key results by the DOI framework

	Site 1	Site 2	Site 3	Site 4
Innovation: compatibility	Patient-level alerts – drug– drug and drug allergy	No patient-level alerts	High priority drug–drug and drug allergy only	Patient-level alerts – drug– drug and drug allergy
Innovation: complexity				All participants recommended alerts that do not impede workflow or are time saving, useful or relevant
Innovation: relative advantage	GFR values, no CKD drug–disease alert	GFR values, no CKD drug–disease alert	GFR values, no CKD drug–disease alert	GFR values, no CKD drug– disease alert
Communication channels	Training	Team meetings with all staff	Email	No standard
Social system	Lead clinic of small multi- clinic organization. Strong role in decision making but must work with the other clinics.	Stand-alone clinic, decision making is all done locally.	Part of large health system. Decision making at higher levels.	Part of large health system. Decision-making at higher levels.

## What this paper adds

- This paper provides guidance in planning to implement electronic drug–disease alerts that warn providers about the use of certain drugs at the time of prescription for patients with specific diseases.
- EHR-based drug–disease alerts must be tailored to the resources, prescribing processes, organizational structures, and communication strategies at the primary care clinics adopting them to maximize acceptance.

## APPENDIX 1 Interview guide

Thank you for agreeing to this telephone interview today as part of our pilot project on improving safe prescribing practices in patients with chronic kidney disease (CKD).

Specifically, we plan to create an EMR alert that will advise providers when they are prescribing drugs that require renal dose adjustment or that are contraindicated in patients with CKD – this alert will be in real time while the prescription is being signed electronically.

1. CKD alert
  1.1. How would you feel about this alert? (Interview introduction explaining the CKD alert intervention omitted in this appendix)
  1.2. What types of barriers might arise during implementation and might prevent full utilization?
  1.3. What would your fellow physicians think about this intervention? What barriers or challenges do you see to this future project, especially at your site?
2. General EMR experience
  2.1. What electronic medical record is available at your practice?
  2.2. What other health software packages do you use (e.g. billing, scheduling and specialty software)?
  2.3. How many years has that electronic medical record been in the practice?
  2.4. Are there any plans to change EMRs in the next 1–3 years?
  2.5. Have there been major changes to your EMR in the past 1–3 years?
  2.6. Who is the current EMR support staff at the practice? What EMR support is available outside of the practice? (e.g. who do you call when you need help with EMR problems?)
  2.7. What are the examples of implementation of EMR-based quality improvement (QI) and research initiatives completed in this clinic in the past 1–2 years? (e.g. examples of when you used your EMR to collect data and improve the quality of care provided in your practice?)
  2.8. For what clinical scenarios does your EMR prompt you to do something (order test, laboratory, med) or not to do something (drug–drug interactions and allergies)? For what processes are EMR alerts available? (define EMR alerts: drug–drug interactions, allergies, disease–drug contraindications and drug dosing) What percentage of the time that you are using an EMR during a patient visit does one of these prompts appear?
  2.9. Is there a particular type of alert that you find to be most useful/least useful?
  2.10. Are there any EMR alerts, flags or decision support tools currently available that specifically relate to patients with CKD? If yes, please describe.
  2.11. What clinical decision support tools (e.g. calculators for dosing and risk assessment [Gail, Framingham]) and links to external evidence-based websites [e.g. NIH, CDC and US Preventive Services Task Force] are available?
  2.12. Does your EMR laboratory result calculate and report glomerular filtration rate?
3. Governance and operational questions
  3.1. Describe how your clinic operates within a larger system (e.g. free standing clinic, part of university system of clinics, part of community clinics, network of private practices)?
  3.2. When changes are implemented to your EMR, can that change be directed specifically to individual providers or individual clinics?
  3.3. By what processes are EMR alerts developed and implemented in the practice?
  3.4. What personnel are required for design, approval and implementation?
  3.5. How are innovative clinical protocols vetted in your institution (e.g. quality committee)? Is there a queue, triage process? By what approval process are they implemented?
  3.6. Are you aware of any evaluation process after the implementation of EMR alerts? Is there an updating process or quality check process after implementation? How have EMR-based initiatives been evaluated at your clinic? Who/what is responsible for this evaluation process?
4. EMR QI/research experience history
  4.1. What CKD-related QI or research initiatives have been completed in the past 5 years at the clinic site?
  4.2. How does the clinic expect to meet the meaningful use objective to implement drug–drug and drug allergy interaction checks (or have they already)?
  4.3. Does your clinic have specific guidelines or protocols or EMR decision support or curricula around CKD safe prescribing?
  4.4. How does your EMR currently identify prescribing safety issues or adverse drug events/prescribing errors/contraindications/dosing issues and relative contraindications (e. g. registry, pharmacist, EMR alert)?
